# Brain metastases in resected non-small cell lung cancer: The impact of different tyrosine kinase inhibitors

**DOI:** 10.1371/journal.pone.0215923

**Published:** 2019-05-02

**Authors:** Po-Jen Yun, Guan-Chyuan Wang, Ying-Yi Chen, Ti-Hui Wu, Hsu-Kai Huang, Shih-Chun Lee, Hung Chang, Tsai-Wang Huang

**Affiliations:** 1 Division of Thoracic Surgery, Department of Surgery, Tri-Service General Hospital, National Defense Medical Center, Taipei, Taiwan, R.O.C; 2 Department of Neurosurgery, Tzu Chi Hospital, Hualien, Taiwan, R.O.C; University of South Alabama Mitchell Cancer Institute, UNITED STATES

## Abstract

**Objectives:**

The purpose of this study was to examine the impact of epidermal growth factor receptor (EGFR) mutation status and tyrosine kinase inhibitors (TKIs) on the survival of brain metastases (BM) in patients with surgically resected non-small cell lung cancer (NSCLC).

**Methods:**

We selected the patients who had developed metastatic NSCLC; analyzed the differences between brain metastases and other sites of metastases, including patient characteristics, EGFR status, and survival; and selected the patients who had BM for further investigation. We also compared the treatment effects of first-generation TKIs with those of second-/third-generation TKIs.

**Results:**

A total of 785 cases of stage I-IIIa NSCLC were reviewed. Thirty-six (4.6%) patients were identified as having BM. Among them, 14 patients had a mutated EGFR status. No association between EGFR mutation and the incidence of BM was observed (p = 0.199). Patients with mutated EGFRs had significantly longer overall survival and post-recurrence survival than patients with wild-type EGFR mutation (p = 0.001 for both). However, there was no survival difference between patients with exon 19 and exon 21 mutations (p = 0.426). Furthermore, patients who received the second- and/or third-generation EGFR-TKIs had better survival than patients who only received first-generation EGFR-TKIs (p = 0.031). A multivariate analysis indicated that the next-generation TKIs (HR, 0.007; 95% CI, 0.000 to 0.556; p = 0.026) and a longer interval before BM development (HR, 0.848; 95% CI, 0.733 to 0.980; p = 0.025) were significant factors in longer survival.

**Conclusions:**

EGFR-TKIs were effective in treating NSCLC patients with BM after curative pulmonary surgery, especially in those patients harboring EGFR mutations. Furthermore, the second-/third-generation EGFR-TKIs showed more promising results than the first-generation EGFR-TKIs in treating those particular patients, though larger studies needed to further prove the results.

## Introduction

The development of brain metastases (BM) is a devastating consequence of disease progression that affects up to 44% of advanced non-small cell lung cancer (NSCLC) patients, particularly patients with adenocarcinoma [1], and indicates treatment failure and worse prognosis. However, NSCLC patients with BM now have a variety of treatment options available, including adjuvant chemotherapy, whole brain radiotherapy (WBRT) with or without stereotactic radiosurgery (SRS), immunotherapy, and epidermal growth factor receptor- tyrosine kinase inhibitors (EGFR-TKIs) for those patients harboring activating EGFR mutations [2].

EGFR-TKIs have been found to be more effective in the treatment of patients with BM than chemo- and/or radiotherapy [3,4]; however, few studies have explored the clinical characteristics, treatment options, and prognoses of NSCLC patients with BM following surgical resection, in spite of the fact that more and more NSCLC patients are currently being diagnosed at the early stage of disease. In addition, patients with BM are especially unique due to their differing intracranial susceptibilities to the different generations of EGFR-TKIs, susceptibilities that are influenced by blood-brain barrier permeability. Previous study had shown that the 1^st^ generation of TKIs had limit blood brain barrier (BBB) penetration. In contrast, the 3^rd^ generation TKI, osimertinib, has better BBB permeability and higher clinical activity than other TKIs [5]. Furthermore, the BBB permeability of gefitinib increases in accordance with escalated dose of radiotherapy [6]. In this study, therefore, we sought to determine some of the distinct characteristics of surgically resected NSCLC patients with subsequent BM, including EGFR features, tumor stages, treatment strategies, and survival. Furthermore, new generations of EGFR-TKIs have been introduced since the first-generation drug, gefitinib (Iressa^®^), was introduced in 2003 and first approved by the FDA to treat NSCLC in August of 2014 [7]. Therefore, we evaluated the effects of different generations of TKIs in treating NSCLC with BM and sought to clarify the prognostic factors for the long-term and post-recurrence survival of patients with BM after complete resection of NSCLC.

## Materials and methods

This study reviewed the database of NSCLC patients who received curative surgery at the Tri-Service General Hospital in Taiwan from July 2004 to July 2017. The institutional Review Board of Tri-Service General Hospital, National Defense Medical Center approved this study and waived individual patient consent. We selected the patients who had developed subsequent BM and analyzed the differences between BM and extracranial metastases, including patient characteristics, EGFR mutation status, and survival. We also compared the treatment effects of first-generation TKIs with those of second-/third-generation TKIs.

For survival analysis, we evaluated the overall survival (OS), disease-free survival (DFS), and survival after brain metastases (SVABM). OS was defined as the interval between the first surgery and the last follow-up or date of death. DFS was defined as the interval between the first surgery and the date of recurrence. Tumor recurrence was confirmed by CT or MRI scan. SVABM was defined as the interval between the identification of BM and the last follow-up or date of death. Factors previously reported to influence the survival of NSCLC were all included in the univariate analysis. These included age, sex, TNM stage, lymphovascular invasion, visceral pleural invasion, EGFR mutation, CEA level, tumor size, ground glass opacity ratio, and surgical removal of the metastatic lesion. Those factors that tended to have impacts on survival were then included in the multivariate calculations.

Univariate categorical variables and univariate continuous variables were analyzed using the chi-square test and Mann-Whitney U test, respectively. The Kaplan–Meier method, log-rank test, and Cox proportional hazard models were used for survival analysis. All the prognostic factors were weighted for significance by hazard ratios, and *P*< 0.05 was considered statistically significant.

## Results

A total of 785 patients with stage I-IIIa NSCLC who all consecutively underwent curative surgery were included. All of the patients underwent comprehensive clinical assessment based on the 7^th^ edition of TNM classification, with those diagnosed before 2009 being restaged according to the new classification. One hundred and fifty-nine of the patients developed metastatic NSCLC and 36 (4.6%) patients were identified as having BM during the period of follow-up, including 19 (53%) men and 17 (47%) women with a median age of 57 years (range: 42 to 86 years). All of the patients were of Asian descent. Among those patients with BM, 21 had a determined EGFR mutation status, and mutated EGFRs were found in 14 (10 had in-frame deletions in exon 19, 2 had exon 21 point mutations, 1 had an exon 21 plus T790M mutation, and 1 had a T790M mutation from an exon 21 point mutation after first-line EGFR-TKI treatment). Among 159 patients with relapsed disease, 87 deaths were recorded, and 10 deaths were recorded in 49 EGFR mutant patients. As for the BM group, 20 patients were died during the follow-up period (including 2 patients with mutated EGFR and 5 patients with wild-type EGFR mutation). Table 1 presents the characteristics of the 36 BM patients, and detailed treatment options after developing BM were described in supplementary data. Some of those patients lacked EGFR mutation status data because they presented to our institution before the EGFR mutation test was adopted as a routine check-up in 2011.

**Table 1:** Clinical characteristics of 36 patients with BM.

	Number	%
**Age, median (range), year**	57 (42–86)	
**Sex**		
Male	19	53
Female	17	47
**Smoking**		
Yes	20	55.6
No	16	44.4
**EGFR mutation**		
Wild type	7	19.4
Exon 19	10	27.8
Exon 20	1	2.8
Exon 21	1	2.8
T790M	2	5.6
NKA	15	41.7
**Number of brain metastases**		
1	13	36.1
≥2	23	63.9
**c-Stage**		
Ia	10	27.8
Ib	5	13.9
IIa	7	19.4
IIb	1	2.8
IIIa	13	36.1
**EGFR-TKIs**		
Gefitinib	12^a^	33.3
Erlotinib	7^b^	19.4
Afatinib	6	16.7
Osimertinib	1	2.8
**Size of primary lung tumor**		
< 2 cm	9	25
≥ 2 cm	27	75
**Tumor histologic type**		
Adenocarcinoma	33	91.7
Large cell carcinoma	2	5.6
Pleomorphic carcinoma	1	2.8

^a^ 4 patients shifted to Erlotinib or Afatinib

^b^ 1 patients shifted to Osimertinib

In comparing the patients with BM to those with other sites of metastases, we found that although the EGFR mutation rate was higher in the BM group, the difference was not statistically significant (p *=* 0.161). There were also no significant differences in sex, TNM stage, T factor, and OS (Table 2) between the two groups. However, we did find that the patients with BM were younger (p = 0.037) and included a higher proportion of patients with advanced N stage (p = 0.024).

**Table 2:** Patient differences in BM vs. other sites of metastases.

	Brain metastases	Other sites of metastases	*P* value
**Age (≤65)**	30 (83.3%)	70 (56.9%)	0.024
**Sex (female)**	17 (47.2%)	59 (48.0%)	0.937
**EGFR mutation rate**	14 (38.9%)	35 (28.5%)	0.161
**TNM stage**			0.425
stage I	15 (41.7%)	71 (57.7%)	
stage II	8 (22.2%)	22 (17.9%)	
stage III	13 (36.1%)	30 (24.4%)	
stage IV	0 (0%)	0 (0%)	
**T stage**			0.986
T1	19 (52.8%)	64 (52%)	
T2	13 (36.1%)	43 (35%)	
T3	3 (8.3%)	11 (8.9%)	
T4	1 (2.8%)	5 (4.1%)	
**N stage**			0.037
N0	18 (50.0%)	89 (72.4%)	
N1	7 (19.4%)	11 (8.9%)	
N2	11 (30.6%)	23 (18.7%)	
N3	0 (0%)	0 (0%)	
**Median overall survival (months)**	58.42	62.13	0.837

**Fig 1:**
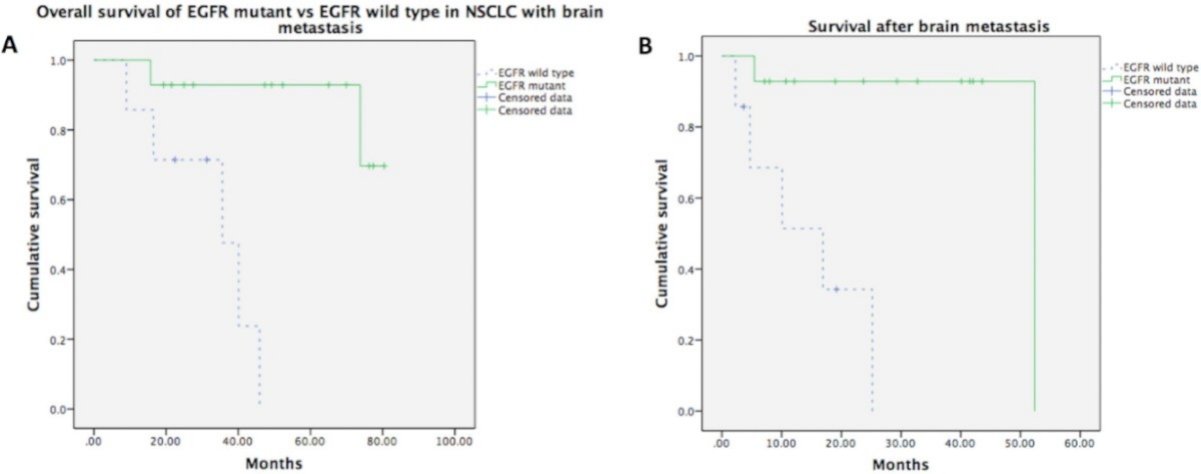
Overall survival and SVABM according to EGFR status. In 36 patients with brain metastases, Fig 1 showed that EGFR-mt patients had better prognosis than EGFR-wt patients on overall survival (p = 0.001) and survival after brain metastasis (p = 0.001).

**Fig 2:**
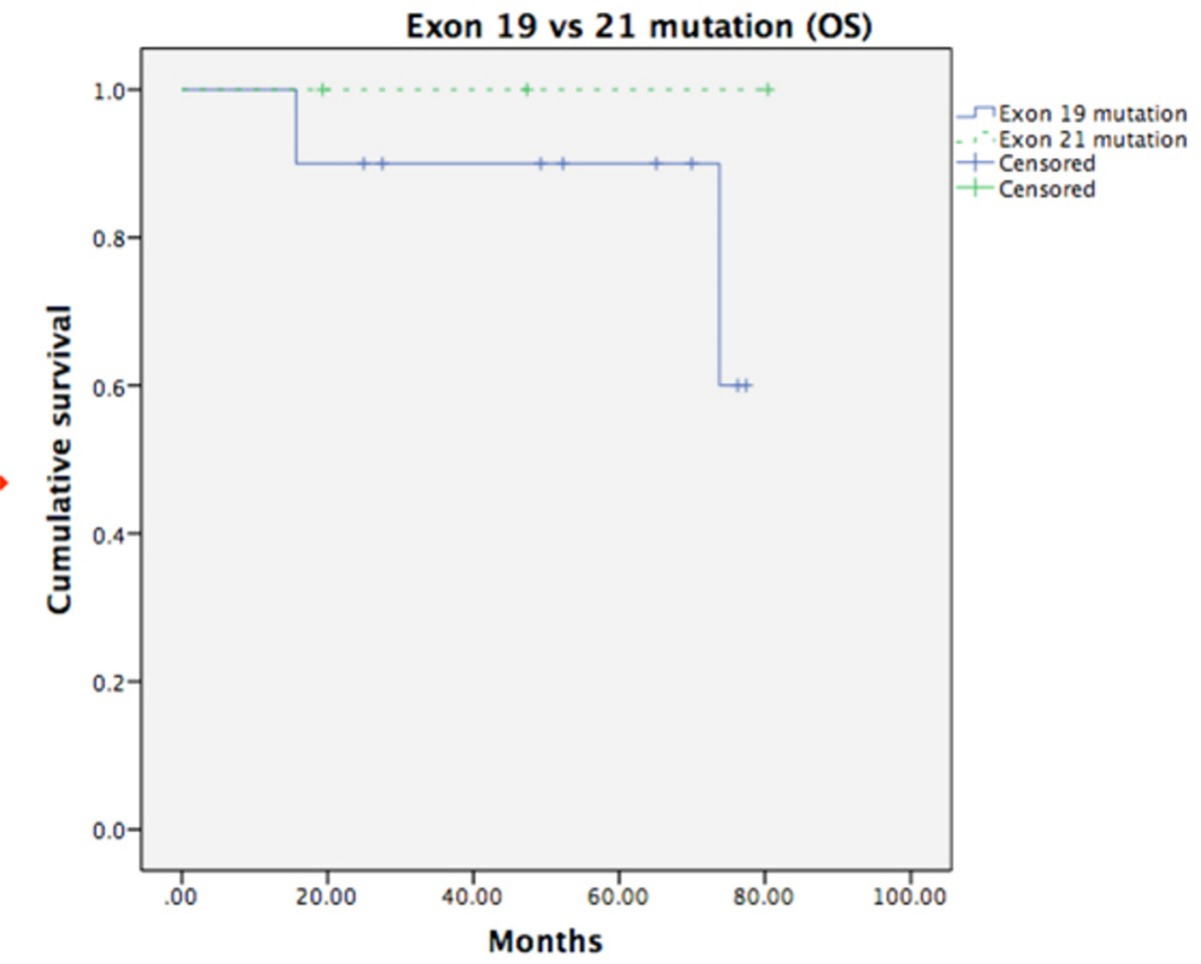
Survival difference between patients with exon 19 and exon 21 mutations. Fig 2 showed that there was no survival difference between exon 19 and exon 21 mutation (p = 0.426).

**Fig 3:**
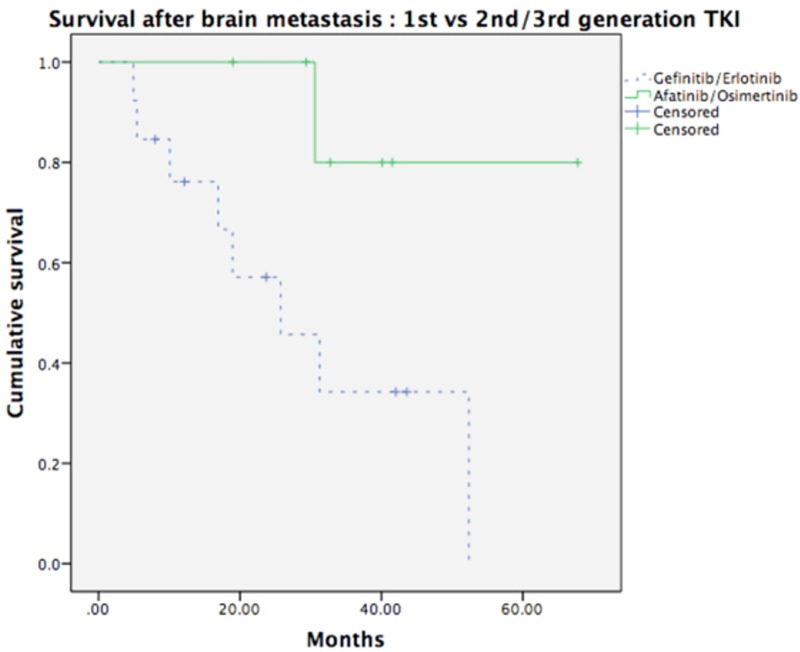
Survival after BM according to different generations of TKIs. In 36 patients with brain metastases, Fig 3 showed the benefit of the second-/third-generation of TKIs on survival after brain metastasis (p = 0.031).

The median follow-up time of the 159 patients who had metastases (including BM and other sites of metastases) was 41.40 months (7.77–149.55 months). Among the patients with BM, the median follow-up period was 41.53 months (range: 8.21–149.55 months), the median OS was 58.42 months, and the median DFS was 17.18 months. Compared to the patients with wild-type EGFR mutation, the patients with mutated EGFR had significantly longer OS (median survival: 35.58 months vs. not reached, p = 0.001) and SVABM (median survival: 52.37 months vs. 16.92 months, p = 0.001) (Fig 1). In addition, we also examined the patients with two common subtypes of EGFR mutation, exon 19 and exon 21 mutations, and found that there was no survival difference between the patients with exon 19 mutations and those with exon 21 mutations (p = 0.426) (Fig 2). As for the effects of treatment with different EGFR-TKIs, we found that the BM patients who had received second- and/or third-generation EGFR-TKIs had better survival than the patients who had only received first-generation EGFR-TKIs (NR vs. 25.76 months, p = 0.031), regardless of whether or not they had received other adjuvant treatments (that is, chemotherapy, radiotherapy, or neurosurgery) (Fig 3).

Univariate analyses demonstrated that female sex, mutated EGFR status, and a longer interval to BM development (that is, DFS) were favorable factors for OS in patients with BM. Furthermore, to confirm which factors were independently associated with better survival, a multivariate analysis was conducted and showed that longer DFS and the use of next-generation (that is, second- and third-generation) TKIs were favorable prognostic factors for OS in patients with BM (HR: 0.883; 95% CI, 0.788–0.991; p = 0.034 and HR: 0.020; 95% CI, 0.001–0.723; p = 0.033, respectively) (Table 3). As for the survival analysis after BM development, the univariate analyses revealed that female sex and mutated EGFR status were related to favorable prognosis, and a multivariate analysis showed that only the use of next-generation TKIs (HR, 0.081; 95% CI, 0.009 to 0.758; p = 0.028) was a significant factor for better survival (Table 4), a finding which was compatible with the results of the OS analysis in surgically treated patients with subsequent BM and indicated that the different generations EGFR-TKIs have a crucial impact on treatment efficacy in this special group of patients.

**Table 3:** Factors associated with overall survival in NSCLC with BM.

	Univariate analysis		Multivariate analysis	
Variables	HR (95% CI)	*P* value	HR (95% CI)	*P* value
**Age**		**0.132**		**0.333**
< 65	1		1	
≥ 65	2.115(0.798–5.605)			
**Sex**		0.040		0.371
Male	1		1	
Female	0.352 (0.141–0.955)		0.404 (0.055–2.948)	
**Stage**		0.907		
I	1			
II	0.923 (0.247–3.450)			
III	1.199 (0.453–3.175)			
**LVI**		0.928		
No	1			
Yes	0.954 (0.343–2.655)			
**VPI**		0.629		
No	1			
Yes	0.607 (0.080–4.600)			
**EGFR**		0.010		
Wild type	1			
Mutated	0.056 (0.006–0.503)			
**Pre-op CEA**		0.757		
≤ 5	1			
> 5	0.851 (0.306–2.367)			
**Tumor size**		0.148		0.478
≤ 2cm	1		1	
> 2cm	2.284 (0.747–6.982)		0.321 (0.014–7.397)	
**GGO ratio**		0.586		
≤ 0.5	1			
> 0.5	1.758 (0.231–13.390)			
**DFS**	0.946 (0.905–0.988)	0.012	0.883 (0.788–0.991)	0.034
**TKI generation**		0.104		0.033
I	1		1	
II/III	0.177 (0.022–1.427)		0.020 (0.001–0.723)	
**Neurosurgery**		0.660		
No	1			
Yes	0.812 (0.321–2.055)			
**Numbers of** **metastases**		0.188		
1 site (BM)	1			
2 sites	2.726 (0.929–7.998)			
3 sites	1.988 (0.471–8.389)			

NSCLC: non-small cell lung cancer; BM: brain metastases; HR: hazard ratio

CI: confident interval; LVI: lymphovascular invasion; VPI: visceral pleural invasion; CEA: carcinoembryonic antigen; GGO: ground-glass opacity

**Table 4:** Factors associated with survival after BM in NSCLC.

	Univariate analysis		Multivariate analysis	
Variables	HR (95% CI)	*P* value	HR (95% CI)	*P* value
**Age**		0.243		0.615
< 65	1		1	
≥ 65	1.785 (0.675–4.720)		0.557 (0.057–5.457)	
**Sex**		0.061		0.305
Male	1		1	
Female	0.405 (0.157–1.044)		0.371 (0.056–2.469)	
**LVI**		0.755		
No	1			
Yes	0.849 (0.305–2.367)			
**VPI**		0.540		
No	1			
Yes	0.531 (0.070–4.014)			
**EGFR**		0.011		
Wild type	1			
Mutated	0.059 (0.007–0.519)			
**DFS**	0.974 (0.934–1.102)	0.224	0.987 (0.922–1.057)	0.709
**Relapse CEA**		0.529		
≤ 5	1			
> 5	1.473 (0.441–4.916)			
**TKI generation**		0.064		0.028
I	1		1	
II/III	0.139 (0.017–1.121)		0.081 (0.009–0.758)	
**Neurosurgery**		0.714		
No	1			
Yes	0.841 (0.333–2.125)			
**Numbers of metastases**		0.177		
1 site (BM)	1			
2 sites	2.365 (0.827–6.763)			
3 sites	3.403 (0.781–14.82)			

## Discussion

In this study, in which we included 785 surgically resected NSCLC patients and identified 36 patients among them who developed postoperative BM, we sought to explore the predictive factors for overall and post-recurrence survival, with a particular focus on EGFR mutation statuses and treatment with different TKIs. The EGFR mutation rate in NSCLC varies according to patient characteristics, including sex and ethnicity. More specifically, it is well known that EGFR mutation incidence is higher in female patients than in male patients, and higher in Asian patients than in Western patients [8]. Midha *et al*. reported the overall frequencies of EGFR mutations were 47% in Asian patients, 15% in European patients, 22% in North American patients, and 36% in South American patients [9]. The patients included in our study were all Taiwanese and were predominantly female (481/785, 61.3%); accordingly, a high EGFR mutation rate could be expected, and indeed, the EGFR mutation rate was 52.3% (265/507) in our patient population. However, our institution did not introduce the EGFR mutation test as a regular examination for surgically treated patients until 2011; thus, a subset of our patients (n = 278) lacked any EGFR mutation data, which probably resulted in a misestimation of the actual EGFR mutation rate.

The association between EGFR mutation status and BM in NSCLC patients has previously been investigated, although no clear conclusions could be drawn from those investigations [10–14], with the molecular aspects of the pathway by which EGFR-mutated cancer cells metastasize remaining unclear to date. In the present study, we obtained results similar to some of previous studies in that the BM patients had a higher EGFR mutation rate compared to the patients with other sites of metastases (38.9% vs. 28.5%). However, this difference between the two groups of patients was not statistically significant (p = 0.161), and there may have been at least a couple of reasons for this lack of significance. First, the patients in this study had a relatively high mutation rate overall (52.3%), which might have reduced the degree of difference between the two groups. Secondly, not all of our patients had EFGR mutation data, and this lack of complete data may have contributed to the results. The patients without EGFR mutation data underwent treatment during a part of the overall study period when the EGFR mutation test was not standard at our hospital. In a previous study, meanwhile, Shin *et al*. reported a significant association between mutated-EGFR status and risk of BM at the time of initial diagnosis, as well as during the follow-up period after curative resection of pulmonary adenocarcinoma [10]. A similar result was also reported in a recent study focused on an Asian population, with that study reporting that NSCLC patients with mutated EGFR had a higher cumulative incidence of subsequent BM, in addition to reporting that further *in vitro* exploration showed that mutated EGFR tumors trigger mesenchymal-like phenotype transformation and induce cancer cell dissemination [11]. In contrast, a retrospective study conducted by Doebele RC *et al*. included 209 consecutive patients with treatment-naïve NCSLC and sought to explore the biological behaviors influenced by the different activations of driver oncogenes, finding that patients with EGFR mutations were more likely to have liver metastases than patients without such mutations [12]. However, no molecular cohort (EGFR, ALK, and KRAS) was observed to have a predisposition to BM, and a recent study comparing EGFR-mutated, KRAS-mutated, and wild-type patients also declared no differences among them in terms of the incidence of subsequent BM [13]. Given that the real molecular aspect explaining the correlation between EGFR mutation status and BM thus remains unclear at present, further clinicomolecular surveys need to be performed in order to draw certain conclusions.

Furthermore, we found that the patients with BM in the current study were younger and more likely to have pN1 and pN2 disease than the patients with extracranial metastases (Table 2). A nomogram to predict BM in curatively resected NSCLC patients developed by Won *et al*. [15] showed that higher pN stage was a significant predictor, in addition to showing that younger age was associated with an increased risk of developing BM, although the association was not statistically significant. In addition, a 3.90-fold higher risk of developing subsequent BM in operable patients with multiple (≥4) metastatic lymph nodes than in those without metastatic lymph nodes was reported by Zhang *et al*. [16]. Our own results in this study regarding the characteristics of subsequent BM in patients with surgically treated NSCLC were consistent with those of these two earlier studies, suggesting that a more strictly surveillance strategy may be appropriate for younger patients and patients who are positive for pathological nodal disease.

One of the latest upgraded prognostic assessments for NSCLC patients with BM was introduced in a study by Sperduto *et al*. [17] that included 2186 patients treated from 2006 to 2014. That study indicated that molecular alternation in EGFR is a favorable prognostic factor in NSCLC patients with BM, a phenomenon that might be derived from the benefit of sensitivity to target therapy in patients with mutated gene status. Another study reported that the standard treatment for multiple BM, whole-brain radiotherapy, could be delayed by the administration of upfront EGFR-TKIs for NSCLC patients with BM, thereby reducing the toxic effect of radiotherapy and improving quality of life for such patients [18]. Moreover, with regard to the treatment of surgically treated NSCLC patients with postoperative disease progression, a Japanese research team conducted a study using the same patient group setting as ours and reported a significantly longer post-recurrence survival of 49 months for EGFR-mutated patients receiving EGFR-TKIs than the 12 months for patients who did not, showing that EGFR-TKIs also contribute to better post-recurrence outcomes [19].

This study could not definitively determine which EGFR mutation subtype resulted in better survival in patients with postoperative BM under EGFR-TKI treatment due to the small sample sizes of patients with the relevant subtypes (though large number of patients were included in the study, only 36 patients had brain metastases), although there was no survival difference evident in our results (Fig 2). A meta-analysis that included patients with advanced NSCLC treated with EGFR-TKIs as first-line therapy was recently conducted [20], with a total of 872 patients with exon 19 deletions and 686 patients with exon 21 L858R substitutions being enrolled, showing that treatment with EGFR-TKIs improves PFS by approximately 50% in those with exon 19 deletions compared to those with exon 21 substitutions. Another previous study also demonstrated poorer PFS with the presence of exon 21 L858R substitutions than exon 19 deletions in patients with advanced NSCLC undergoing erlotinib treatment [21]. The molecular associations between different EGFR mutations and outcomes under EGFR-TKI treatment are still unclear; however, these results indicate that the further development of EGFR-TKI antitumor effects against tumors harboring exon 21 mutations remains important.

Based on the results of previous clinical trials [22–27], EGFR-TKIs including gefitinib, erlotinib, and afatinib are currently regarded as the first-line treatment for patients with EGFR-mutated tumors. This conclusion is based on the associated survival benefit (that is, in terms of PFS) versus chemotherapy in the advanced disease stage. Two randomized controlled trials have shown that gefitinib significantly increases PFS when compared to chemotherapy. The WJTOG3405 study reported a PFS of 9.2 months for patients treated with gefitinib and 6.3 months for patients treated with chemotherapy [22]. The other study also reported a significantly longer PFS in a gefitinib group (10.8 months) than in a chemotherapy group (5.4 months) [23]. In a similar way, two randomized controlled trials have shown that erlotinib significantly prolongs PFS when compared to chemotherapy, with the PFS of the erlotnib groups ranging from 9.7 months (EURTAC study) to 13.1 months (OPTIMAL study) [24,25].

The LUX-Lung clinical trials further focused on a second-generation EGFR-TKI, afatinib, performing a comprehensive survey of treatment effects in patients with advanced EGFR-mutated NSCLC. A subgroup analysis of the LUX-Lung 3 and 6 results demonstrated a significantly better PFS in the setting of first-line afatinib versus chemotherapy in patients with BM (8.2 months versus 5.4 months; HR: 0.50; p = 0.0297) [4]. Furthermore, the LUX-Lung 7 study revealed a significantly improved PFS (11.0 months versus 10.9 months; HR: 0.73; 95% CI, 0.57–0.95; p = 0.017) and time-to-treatment failure (13.7 months versus 11.5 months; HR: 0.73; 95% CI, 0.58–0.92; p = 0.0073) in patients with EGFR-mutated NSCLC treated with afatinib as compared to gefinitib. Our study results also revealed a similar tendency, with the patients with BM who had received second- and/or third-generation EGFR-TKIs having better survival than those who had only received first-generation EGFR-TKIs. The enhanced antitumour ability of afatinib revealed in this study might reflect its irreversible inhibition of EGFR signaling [28].

In our database, there was one patient (no.10) who sustained long-term survival under osimertinib treatment after developing a T790M mutation under erlotinib treatment (41.5 months after the diagnosis of BM). The abilities of the different generations of EGFR-TKIs to penetrate the blood-brain barrier (BBB) may directly reflect their different intracranial antitumor effects. That said, while the penetration ability of gefitinib was previously reported to be approximately 1% of serum level and that of erlotinib was previously reported to range from 2.5% to 13%, very little is known about the BBB penetration ability of afatinib [29]. Nevertheless, the survival benefit in BM patients shown in this study suggests that the CSF dose of afatinib was sufficient to inhibit tumor progression. Furthermore, in recent animal studies, a promising result of greater BBB penetration was noted for the third-generation EGFR-TKIs osimertinib (AZD9291) and AZD3759 in comparison to gefitinib, erlotinib, and afatinib [5,30]. These results showed that newly developed EGFR-TKIs can achieve better intracranial antitumor activities while avoiding intolerable toxicities outside the CNS system.

The present study had some limitations. First, it was a retrospective study of a single center with a relatively small number of patients, and some of the patients lacked EGFR mutation data. Second, further EGFR mutation surveys of surgical brain tissue should be conducted to clarify the exact molecular changes in metastatic lesions, given that genetic heterogeneity between the primary tumor and CNS metastases may exist. Third, the inconsistent treatment strategies used for postoperative BM patients may obscure the real intracranial antitumor effects of EGFR-TKIs. Previous studies have shown that upfront radiotherapy will disrupt BBB tight junctions and cause elevated drug concentrations in the CSF; thus, a study with a unified post-recurrence treatment setting should be conducted to eliminate this bias. Finally, we did not include the functional status of patients in our analyses. Patients with better functional status were more likely to receive aggressive therapy, whereas patients with poorer functional status tended to be treated more conservatively. Nevertheless, our study focused on patients of East Asian ethnicity who underwent curative pulmonary surgery, with the results providing the clinical implication that more strictly brain surveillance should be undertaken for younger patients and patients with positive pN disease, and next-generation of EGFR-TKIs should be strongly considered for the treatment of NSCLC patients with subsequent BM.

## Supporting information

S1 Table EGFR status and detailed treatments of the 36 patients with BM. (DOCX)

S1 Fig Flow chart of the cases analyzed. (TIFF)

S2 Fig The letter of approval from Institutional Review Board (IRB) and institutional ethics committee. (TIFF)

S1 Dataset The minimal data set, for the manuscript results presented here, is attached. (XLSX)
