# Supplementary material for: Brain metastases in resected non-small cell lung cancer: The impact of different tyrosine kinase inhibitors
Source: PLoS One. 2019 May 2;14(5):e0215923. doi: 10.1371/journal.pone.0215923 (PMC6497246; doi:10.1371/journal.pone.0215923)
Supplement: S1 Table — (DOCX) [file pone.0215923.s001.docx]

**S1 Table. EGFR status and detailed treatments of the 36 patients with BM**

| **No** | **Sex** | **Age** | **Stage** | **EGFR status** | **Treatment after BM** | **Status** |
| --- | --- | --- | --- | --- | --- | --- |
| 1 | M | 61 | IIIA | Wild type | Tegafur+ Uracil (UFUR®); WBRT, SRS | Alive |
| 2 | M | 58 | IIIA | Exon 19 | WBRT | Alive |
| 3 | M | 64 | IIIA | Nil | Phyxol (Paclitaxel®), Cisplatin (Abiplatin®); Neurosurgery | Dead |
| 4 | M | 86 | IA | Wild type | Vinorelbine (Navelbine®), Pemetrexed (Alimta®) | Dead |
| 5 | F | 70 | IIA | Exon 19 | Erlotinib (Tarceva®) | Alive |
| 6 | F | 65 | IA | Nil | Gemcitabine (Gemzar®), Cisplatin (Abiplatin®); WBRT | Dead |
| 7 | F | 49 | IIIA | Exon 19 | Gefitinib (Iressa®), Pemetrexed (Alimta®), Cisplatin (Abiplatin®), Docetaxel (Taxotere®); WBRT | Dead |
| 8 | F | 47 | IIIA | Exon 19 | Gefitinib (Iressa®), Pemetrexed (Alimta®), Cisplatin (Abiplatin®); WBRT | Alive |
| 9 | M | 56 | IA | Wild type | Pemetrexed (Alimta®), Cisplatin (Abiplatin®), Erlotinib (Tarceva®), Phyxol (Paclitaxel®) | Dead |
| 10 | M | 53 | IA | T790M/Exon 21 | Pemetrexed (Alimta®), Gemcitabine (Gemzar®), Erlotinib (Tarceva®), Docetaxel (Taxotere®), Osimertinib (Targrisso®) | Alive |
| 11 | F | 54 | IB | Nil | Afatinib (Giotrif ®) | Dead |
| 12 | F | 49 | IA | Nil | Gemcitabine (Gemzar®), Paraplatin (Carboplatin®), Gefitinib (Iressa®); WBRT, Neurosurgery | Dead |
| 13 | M | 62 | IB | Nil | Tegafur+ Uracil (UFUR®); SRS | Dead |
| 14 | M | 60 | IA | Nil | Pemetrexed (Alimta®), Gefitinib (Iressa®); WBRT, SRS | Dead |
| 15 | M | 74 | IIIA | Nil | Cisplatin (Abiplatin®), Vinorelbine (Navelbine®), Gefitinib (Iressa®) | Dead |
| 16 | M | 43 | IIIA | Wild type | Docetaxel (Taxotere®), Gefitinib (Iressa®), Cisplatin (Abiplatin®), Gemcitabine (Gemzar®); WBRT | Dead |
| 17 | M | 42 | IIA | Exon 20 | Cisplatin (Abiplatin®), Vinorelbine (Navelbine®); Neurosurgery | Alive |
| 18 | F | 53 | IIIA | T790M/Exon 21 | Gefitinib (Iressa®), Erlotinib (Tarceva®) | Alive |
| 19 | F | 50 | IA | Exon 19 | Gefitinib (Iressa®), Pemetrexed (Alimta®), Cisplatin (Abiplatin®), SRS | Alive |
| 20 | F | 50 | IB | Nil | Phyxol (Paclitaxel®), Cisplatin (Abiplatin®); WBRT, Neurosurgery | Alive |
| 21 | M | 81 | IIA | Wild type | Vinorelbine (Navelbine®); SRS | Alive |
| 22 | F | 63 | IIA | Exon 19 | Cisplatin (Abiplatin®), Vinorelbine (Navelbine®), Afatinib (Giotrif®); WBRT | Alive |
| 23 | M | 64 | IB | Exon 21 | Vinorelbine (Navelbine®), Afatinib (Giotrif®); Neurosurgery | Alive |
| 24 | F | 43 | IIA | Exon 19 | Gefitinib (Iressa®), Afatinib (Giotrif®); SRS, Neurosurgery | Alive |
| 25 | F | 63 | IIIA | Nil | Gemcitabine (Gemzar®), Cisplatin (Abiplatin®), Docetaxel (Taxotere®) | Dead |
| 26 | M | 68 | IIA | Nil | WBRT | Dead |
| 27 | M | 56 | IIIA | Exon 19 | Afatinib (Giotrif®); SRS, Neurosurgery | Alive |
| 28 | F | 60 | IIB | Nil | Erlotinib (Tarceva®); WBRT | Dead |
| 29 | M | 53 | IIA | Wild type | Cisplatin (Abiplatin®), Vinorelbine (Navelbine®), Pemetrexed (Alimta®), Nivolumab (Opdivo®) | Dead |
| 30 | F | 47 | IB | Exon 19 | Gefitinib (Iressa®), Erlotinib (Tarceva®), Cisplatin (Abiplatin®), Pemetrexed (Alimta®); WBRT | Alive |
| 31 | M | 72 | IIIA | Nil | Phyxol (Paclitaxel®), Cisplatin (Abiplatin®), Vinorelbine (Navelbine®); SRS, Neurosurgery | Dead |
| 32 | M | 56 | IA | Nil | Docetaxel (Taxotere®), Gemcitabine (Gemzar®); SRS | Dead |
| 33 | M | 50 | IIIA | Nil | Neurosurgery, WBRT | Dead |
| 34 | F | 59 | IIIA | Nil | Pemetrexed (Alimta®), Erlotinib (Tarceva®), Phyxol (Paclitaxel®), Afatinib (Giotrif®), Gemcitabine (Gemzar®); WBRT | Alive |
| 35 | F | 53 | IA | Wild type | Cisplatin (Abiplatin®), Pemetrexed (Alimta®) | Dead |
| 36 | F | 65 | IA | Exon 19 | Gefitinib (Iressa®); WBRT | Dead |

WBRT: whole-brain radiation therapy; SRS: stereotactic surgery
