# Supplementary figures and images for: Brain metastases in resected non-small cell lung cancer: The impact of different tyrosine kinase inhibitors
Source: PLoS One. 2019 May 2;14(5):e0215923. doi: 10.1371/journal.pone.0215923 (PMC6497246; doi:10.1371/journal.pone.0215923)

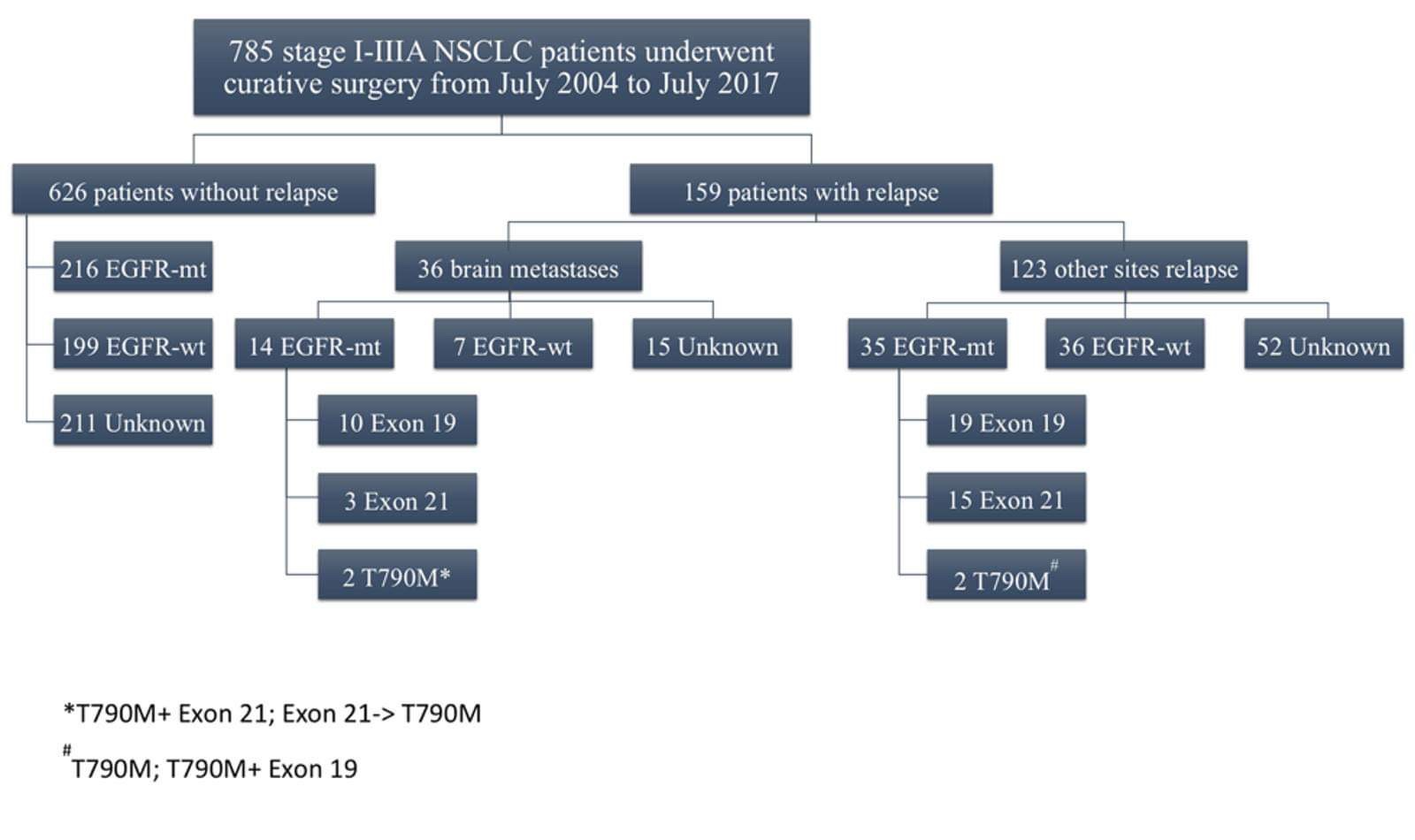

Supplement: S1 Fig — (TIFF) [file pone.0215923.s002.tiff]

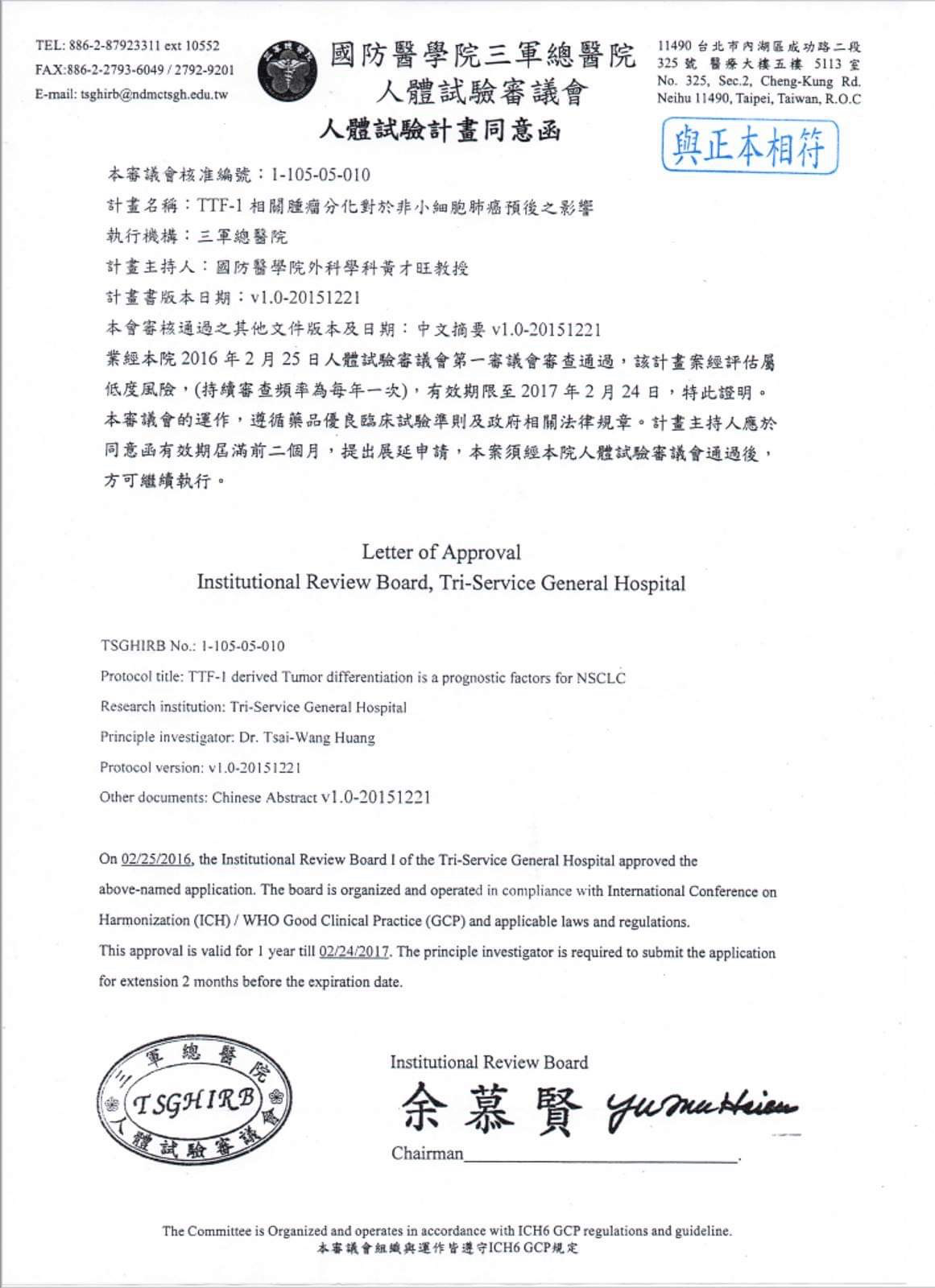

Supplement: S2 Fig — (TIFF) [file pone.0215923.s003.tiff]
